# Development of Small-Molecule Inhibitors Against Zika Virus Infection

**DOI:** 10.3389/fmicb.2019.02725

**Published:** 2019-12-06

**Authors:** Lili Wang, Ruiying Liang, Yaning Gao, Yanbai Li, Xiaoqian Deng, Rong Xiang, Yina Zhang, Tianlei Ying, Shibo Jiang, Fei Yu

**Affiliations:** ^1^Research Center of Chinese Jujube, Hebei Agricultural University, Baoding, China; ^2^College of Life and Science, Hebei Agricultural University, Baoding, China; ^3^Department of Natural Medicines, School of Pharmaceutical Sciences, Peking University, Beijing, China; ^4^MOE/NHC/CAMS Key Laboratory of Medical Molecular Virology, School of Basic Medical Sciences, Shanghai Medical College, Fudan University, Shanghai, China

**Keywords:** Zika virus, life cycle, small-molecule inhibitor, treatment, mechanism

## Abstract

In recent years, the outbreak of infectious disease caused by Zika virus (ZIKV) has posed a major threat to global public health, calling for the development of therapeutics to treat ZIKV disease. Here, we have described the different stages of the ZIKV life cycle and summarized the latest progress in the development of small-molecule inhibitors against ZIKV infection. We have also discussed some general strategies for the discovery of small-molecule ZIKV inhibitors.

## Introduction

**FIGURE 1:**
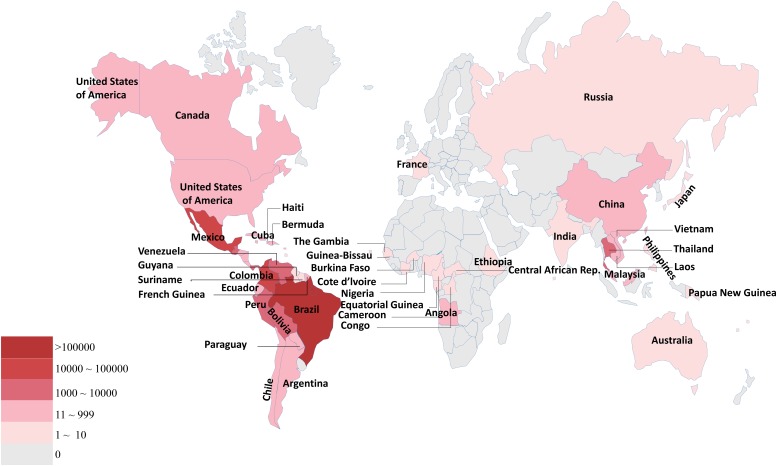
Summary of ZIKV morbidity statistics with country- and quarter-level panel data. The deeper the color, the higher the number of infected people.

Zika virus (ZIKV) is an arthropod-borne virus (arbovirus) belonging to the family Flaviviridae and genus Flavivirus. As a single-stranded positive RNA virus, the genome of ZIKV is approximately 10 kb and encodes three structural proteins and seven non-structural proteins (Wang et al., 2016). In 1947, ZIKV was discovered and isolated from a sentinel Rhesus monkey in the Zika Forest of Uganda (Dick et al., 1952). However, it was only in 2015 that the first outbreak of ZIKV-caused diseases was reported in Brazil (Petersen et al., 2016) with more than one million cases. Since then, it rapidly spread to 84 countries around world, particularly in South America, rendering ZIKV a public health threat (Han and Mesplede, 2018; Figure 1).

Mild symptoms include fever, rash, headache, and joint pain, but the major concern involves the potential for severe neurological disorders, such as microcephaly, neurological disorders in newborns, meningo-encephalitis, Guillain–Barré syndrome, myelitis, and ocular abnormalities (Barros et al., 2018). Until now, neither a specific antiviral drug nor a vaccine has been developed to prevent or cure ZIKV infection. However, several well-characterized drug targets encoded by the virus, or presented in host cells, may help us prevent or treat ZIKV infection. In this review, we focus on current progress on the research and development of small-molecule ZIKV inhibitors, either viral or host cell inhibitors, targeting different stages of the ZIKV life cycle. Such data are essential to the design of drugs and drug delivery modalities against ZIKV and related viruses.

## ZIKV Life Cycle and Potential Targets for the Development of Small-Molecule Inhibitors Against ZIKV Infection

The life cycle of ZIKV can be divided into four stages, including virus entry, genome replication, virus assembly, and release. Mature ZIKV particles first adhere to host cells by interacting with specific receptors on host cells, such as DC-SIGN, AXL, Tyro, and TIM-1 (Musso and Gubler, 2016; Nowakowski et al., 2016; Meertens et al., 2017). Several proteins, including DC-SIGN and TIM as well as some TAM proteins that belong to the phosphatidylserine receptor family, have been reported to act as receptors for entry of dengue virus (DENV)(Lozach et al., 2005; Meertens et al., 2012; Perera-Lecoin et al., 2013). To determine whether these receptors are also involved in ZIKV entry, a series of transfected HEK293T cells expressing DC-SIGN, TIM-1, or a TAM family member (AXL or Tyro3) could be infected by ZIKV at varying degrees (Hamel et al., 2015). DC-SIGN consists of group II (calcium-dependent with single carbohydrate recognition domain) transmembrane C-type lectins that can interact through their carbohydrate recognition domains to bind carbohydrates to viral protein E (Zelensky and Gready, 2005; Cruz-Oliveira et al., 2015). DC-SIGN also plays an important role in flavivirus binding and the infection of myeloid cells (Navarro-Sanchez et al., 2003) as it mediates attachment of viral particles on the cell surface and facilitates their interaction with primary receptors on the host cell (Chen et al., 1997; Germi et al., 2002). Tyro3 and AXL belong to the TAM family, a group of three receptor protein tryrosine kinases that mediate the clearance of apoptotic cells (Lemke and Rothlin, 2008). AXL is expressed in astrocytes and microglia in the human brain development and mediates ZIKV infection of glial cells (Nowakowski et al., 2016; Meertens et al., 2017). AXL consisting of two different Gas6-binding epitopes, including the N-terminal Ig-like domain, and a second Ig domain exists in the dimeric form. Gas6, which is the ligand of AXL, connects ZIKV to glial cells. TIM-1, which is abundant on Th-2 T cells, mucosal epithelial cells, and mast cells, mediates the attachment of ZIKV particles on the cell surface to facilitate their interaction with AXL as well as the subsequent infection (Hamel et al., 2015). The availability of different entry receptors is likely to provide an evolutionary advantage for the virus, and, as a result, the virus is able to infect a wide range of human host cells.

**FIGURE 2:**
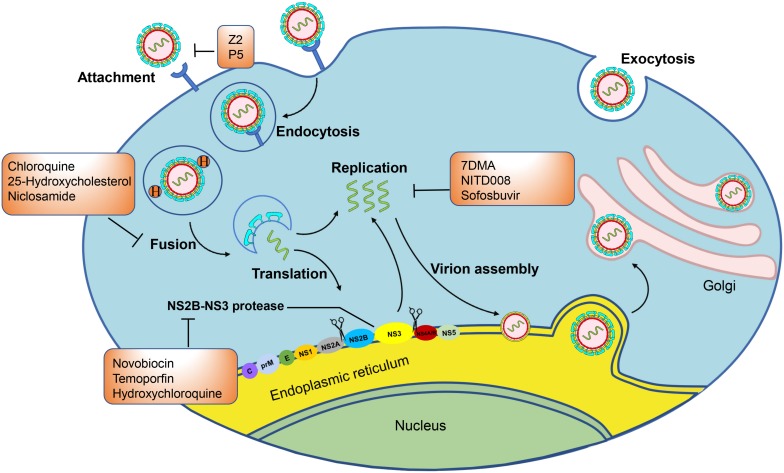
Zika virus infectious life cycle: Host cell membrane receptors bind the E protein of the mature Zika virion, triggering endocytosis. The acidic environment of the endosome induces fusion of the host endosome membrane with the viral envelope and the release of the RNA genome. The RNA is translated into a polyprotein complex, which is cleaved by the host and viral proteases in the ER lumen and cytoplasm, respectively. Following translation, a replication complex is assembled and associated with virus induced membranes where viral replication takes place. The methylated (+) ssRNA, C, E, and prM proteins assemble to form immature virions in the endoplasmic reticulum (ER). The immature virions bud out of the ER into the Golgi apparatus, and they then mature in the trans-Golgi network that are released by exocytosis. In the orange boxes are the names of the compounds that can inhibit the marked steps of the virus lifecycle and that are evaluated in animal models or clinical trials.

After binding with host cells, ZIKV is internalized by clathrin-mediated endocytosis and traffics to Rab5^+^ endosomes (Wang X. et al., 2017; Mottin et al., 2018). In the process of entering the host cell, AXL kinase activity is activated by the ZIKV/Gas6 complex, which downregulates interferon signaling and promotes infection. Then, the endosome membrane and virus envelope (E) are fused under the acidic environment of the endosome. The viral genomic RNA is then released into the cytoplasm (Wang X. et al., 2017; Mottin et al., 2018). In the process of virus entry, some inhibitors can block viral attachment, endocytosis, and fusion. The proteins E on ZIKV and the DC-SIGN, AXL, Tyro, and TIM-1 entry/adhesion factors on the host cell are involved in viral attachment, endocytosis, fusion, and entry (Hasan et al., 2017; Heinz and Stiasny, 2017; Shi and Gao, 2017). They all therefore serve as targets for the development of small molecule inhibitors. After virus entry, the genome of ZIKV is translated and cleaved into three structural proteins, including Capsid (C), Precursor of the membrane protein (prM)/membrane protein (M), Envelope (E), as well as seven non-structural proteins (NS1, NS2A, NS2B, NS3, NS4A, NS4B, and NS5). The NS1 protein is related to flavivirus replication and virion maturation. The NS2B protein activates the active region of the NS3 protein and forms an NS2B-NS3 complex with the NS3 protein to exert proteolytic enzyme activity. The NS4A and NS4B proteins comprise the endoplasmic reticulum (ER)-associated replication complex. The NS5 protein contains the C-terminal RNA-dependent RNA polymerase domain and the N-terminal methyltransferase domain, which cooperate during the initiation and extension of RNA synthesis. In addition, the NS5 protein is the largest NS protein in molecular weight and the most highly conserved. Then RNA is replicated with the actions of NS1, NS2B-NS3 proteinase and NS3 helicase, NS5 methyltransferase, and NS5RdRp. Viruses encode their own essential proteases in the viral replication process, which can serve as targets for therapeutic intervention. The methylated (+) ssRNA, C, E, and prM proteins are assembled to form immature virions in the ER. Then, the ER vesicles transport the virus particles to Golgi apparatus, and the virus particles undergo surface polysaccharide modification, prM-E protein trimer rearrangement, and Furin protease cleavage prM while mature virus particles with a smooth surface are produced, finally leaving the host cell by exocytosis as mature virus (Figure 2; Wang X. et al., 2017; Mottin et al., 2018). Some small molecule inhibitors that target the C protein or inhibit viral capsid formation are able to affect viral assembly and release. Meanwhile, correct expression and processing of nascent proteins in host cells are essential for efficient viral replication. Several host proteins, such as ER membrane complex, α-glucosidase, cyclophilin, and proteasome elements, are responsible for monitoring proper protein synthesis, folding, and degradation. Impairment of these functions results in reduced viral assembly and budding. Therefore, these host proteins may also serve as targets for the development of small molecule ZIKV inhibitors.

Most antiviral drugs are small-molecule inhibitors that target different stages of the viral life cycle by interacting with virus or host proteins critical for virus replication (De Clercq and Li, 2016). For example, inhibiting AXL function can protect cells from infection and, thus, may be a potential target for the production of entry inhibitors. However, destroying AXL function may also have many adverse consequences (Nowakowski et al., 2016). In addition, the proteases crucial for ZIKV replication are potential targets for developing ZIKV replication inhibitors. Therefore, more effective and appropriate targets need to be developed by researchers.

## Current Small-Molecule Inhibitors Against ZIKV Infection and Their Mechanisms of Action

### ZIKV Entry Inhibitors

#### ZIKV Inhibitors Targeting Viral E Protein

The structure of ZIKV envelope protein (E protein) is similar to that of other flaviviruses, and it has three characteristic domains: a central β-barrel-shaped domain I, a Slender finger-like domain II, and a C-terminal immunoglobulin-like domain III (Dai et al., 2016). The recognition and binding of ZIKV E proteins to host cell receptors mark the beginning of ZIKV infection; therefore, some inhibitors designed for envelope proteins can effectively inhibit virus infection (Byrd et al., 2013).

**FIGURE 3:**
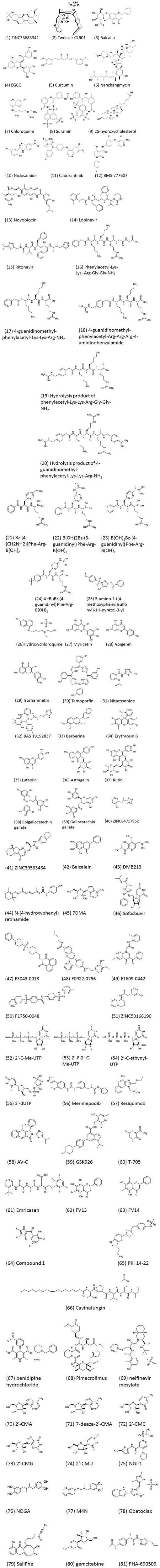
Chemical structures of ZIKV small-molecule inhibitors.

Small molecule inhibitors that specifically target the ZIKV E protein have been reported. Peptide Z2 (Table 1), derived from the stem region of the ZIKV E protein, inhibits vertical transmission of ZIKV in pregnant C57BL/6 mice and protects type I or type I/II interferon receptor-deficient mice against lethal ZIKV challenge (Yu et al., 2017). Peptide Z2 can interact with viral E proteins to form a membrane pore and disrupt the integrity of the viral membrane (Yu et al., 2017). ZINC33683341 [Figure 3(1); Fernando et al., 2016], which can bind with the ZIKV E protein, is preferential when compared with glycan and can block the formation of glycoside bonds between ZIKV and host Vero cells at the concentration of 100 μM (Table 2; Fernando et al., 2016).

**TABLE 1:** Zika virus peptide inhibitors.

**Inhibitor**	**Sequence**	**Testing model**	**Cell lines**	**IC_50_ (μM)**	**CC_50_ (μM)**	**References**
Peptide Z2	MAVLGDTAWDF	*in vitro*	BHK21 cells	1.75 ± 0.13	–	Yu et al. (2017)
	GSVGGALNSLG			3.69 ± 0.27		
	KGIHQIFGAAF		Vero cells			
P5	GQASNGVFVIH	*in vitro*	Vero cells	3.27	–	Chen et al. (2017)
	WGKFDSFGIAV					
Ev37	GLINEKKVQQYLDE	*in vitro*	Huh-7 cells	–	–	Li et al. (2019)
	KLPNGVVKGALKSL					
	VHKAAKNQNLCAF					
	NVDTVGMCDADCK					
	RQGKAKGVCHGT					
	KCKCDVELSYKK					
Aprotinin	–	*in vitro*	–	0.07 ± 0.012	–	Shiryaev et al. (2017)
Acyl-KR-aldehyde	–	–	–	0.208	–	Li et al. (2017d)
AH-D peptide	SGSWLRDVWDWIC	*in vivo*	In mouse	0.0119	–	Cho et al. (2009), Jackman et al. (2018)
	TVLTDFKTWLQSKL					
FFAAP	FFAAP	*in vitro*	Human JEG-3 cells Vero cells		–	Margueron and Reinberg (2011), Di Croce and Helin (2013), Kim et al. (2015), Vasireddi et al. (2019)
ZMP STE24	–	*in vitro*	T98-G cells	–	–	Fu et al. (2017)

**TABLE 2:** Zika virus entry inhibitors.

**Number of chemical structures in Figure 3**	**Inhibitor**	**Testing model**	**Cell lines**	**IC_50_ (μM)**	**CC_50 (_μM)**	**References**
1	Tweezer CLR01	*in vitro*	Vero E6 cells	8.2	–	Bavari et al. (2002), Chazal and Gerlier (2003), Brugger et al. (2006), Lorizate et al. (2013), Rocker et al. (2018)
2	ZINC33683341	*in vitro*	Vero cells	–	–	Fernando et al. (2016)
3	Baicalin	*in vitro*	Vero cells	14	553	Oo et al. (2019)
4	EGCG	*in vitro*	Vero E6 cells	–	–	Song et al. (2005), Isaacs et al. (2008), Nance et al. (2009), Calland et al. (2012), Carneiro et al. (2016)
5	Curcumin	*in vitro*	Huh-7 cells	1.90	11.6	Song and Ni (1998), Mounce et al. (2017), Nitsche et al. (2017), Roy et al. (2017)
6	Nanchangmycin	*in vitro*	Human U2OS cells	0.1	7	Liu et al. (2008), Pierson and Kielian (2013), Rausch et al. (2017)
			Human HBMEC cells	0.4	>10	
			Human JEG-3 cells	0.97	6.10	
7	Chloroquine	*in vitro*	Huh-7 cells	1.72	–	Li et al. (2017b)
		*in vivo*	Vero cells; BALB/c mice and A129 mice	4.15		
8	Suramin	*in vitro*	Vero cells	39.8	1900	Albulescu et al. (2017), Coronado et al. (2018)
9	25-hydroxycholesterol	*in vitro* *in vivo*	Vero cells; BALB/c mice and A129 mice	0.188	–	Li et al. (2017a)
10	Niclosamide	*in vitro*	A549 cells SNB-19 cells	12.3 ± 0.6 1.72	4.8 ± 1.0	Baell and Holloway (2010), Li et al. (2017d), Xu et al. (2016), Li et al. (2017d)
11	Cabozantinib	*in vitro*	Human U2OS cells	0.2	>10	Rausch et al. (2017)
12	BMS-777607	*in vitro*	Human U2OS cells	0.6	>10	Rausch et al. (2017)

Some small molecule inhibitors that non-specifically target the ZIKV E protein were also active against other medically relevant viruses that use a similar route of entry. P5, a peptide extracted from the stem of Japanese encephalitis virus E protein, can inhibit ZIKV entry into host cells by changing the conformation of the E protein under low pH. The hydrophobicity of the last seven amino acid residues is also considered to be the key to the binding of the viral membrane (Table 1; Chen et al., 2017). *In vivo* experiments in mice highly sensitive to ZIKV showed that P5 can inhibit spermatic tubule disorder and reproductive epithelial cell degeneration while also alleviating the circulatory constriction of blood vessels (Chen et al., 2017).

The molecular tweezer CLR01, which has potent inhibition activity to envelope viruses, can inhibit ZIKV strains in Vero E6 cells [Figure 3(2) and Table 2] by destroying the intact membrane structure that is enriched with high levels of sphingolipid and cholesterol (Bavari et al., 2002; Chazal and Gerlier, 2003; Brugger et al., 2006; Lorizate et al., 2013; Rocker et al., 2018). In addition, it can inhibit ZIKV infection in semen, urine, saliva, cerebrospinal fluid, and other body fluids, but lose activity in serum (Rocker et al., 2018). Some studies have attributed this effect to the relatively high protein content in serum (Rocker et al., 2018).

Baicalin [Figure 3(3)], which has high affinity to the virus E protein and low toxicity to cells, can inhibit ZIKV from entering cells (Table 2; Oo et al., 2019). (-)-Epigallocatechin gallate (EGCG), a polyphenol from green tea, was shown to inhibit many viruses [Figure 3(4) and Table 2; Isaacs et al., 2008; Nance et al., 2009; Calland et al., 2012]. Accordingly, EGCG can bind to the ZIKV E protein to block ZIKV entry into host cells (Song et al., 2005). However, EGCG contains the catechol group that may non-specifically inhibit many different targets (Mottin et al., 2018). Curcumin can inhibit ZIKV infection in a dose-dependent manner [Figure 3(5)]. It is not only a replication inhibitor of ZIKV, but also prevents the viral E protein from binding to the cell surface (Mounce et al., 2017; Roy et al., 2017). In Vero cells, the IC_50_ and CC_50_ value of curcumin inhibiting ZIKV is 1.90 and 11.6 μM, respectively (Table 2; Mounce et al., 2017). Nanchangmycin [Figure 3(6)], produced by Streptomyces nanchang fermentation, can inhibit gram-positive bacteria and has insecticidal and antibacterial activities against poultry *in vitro* (Rausch et al., 2017). For Zika virus, Nanchangmycin can inhibit ZIKV infection by blocking clathrin-mediated endocytosis with IC_50_s between 0.1 and 0.4 μM, and it has low toxicity in this range (Table 2) in human U2OS cells, human brain microvascular endothelial cells (HBMEC), and human Jeg-3 cells, respectively (Rausch et al., 2017).

#### ZIKV Inhibitors Targeting Endosome

Endosomes provide a transport route for ZIKV to enter host cells. Ev37 (Table 1), an endosomal scorpion peptide inhibitor, can effectively inhibit ZIKV infection at a non-cytotoxic concentration (Li et al., 2019). Ev37 is a broad-spectrum and specific antiviral peptide, which can alkalize the pH value of endosomes, inhibit the release of a viral genome, and prevent it from entering the cytoplasm, thus blocking ZIKV infection (Li et al., 2019). In Huh-7 cells, Ev37 can reduce 87% of ZIKV infection at a concentration of 10 μM (Li et al., 2019). Chloroquine (Li et al., 2017a), Suramin (Albulescu et al., 2017), and 25-hydroxycholesterol [Figure 3(7–9) and Table 2; Li et al., 2017a) demonstrated their ability to inhibit ZIKV internalization *in vitro*. Niclosamide is an FDA-approved drug broadly used in the treatment of intestinal helminthiasis [Figure 3(10) and Table 2]. It can prevent endosomal acidification, but the mechanism is not fully elucidated (Fonseca et al., 2012; Jurgeit et al., 2012).

#### ZIKV Inhibitors Targeting AXL

AXL is a tyrosine kinase receptor (TKR), which can mediate viral attachment to host cells. Therefore, it is necessary to inhibit primary cells with high AXL content (Nowakowski et al., 2016). Cabozantinib and BMS-777607 are two kinase inhibitors that inhibit AXL [Figure 3(11,12); Rausch et al., 2017]. In human U2OS cells, their IC_50_ values are 0.2 and 0.6 μM, respectively, and the CC_50_ values are greater than 10 μM (Table 2; Rausch et al., 2017). However, the experiments showed that AXL inhibitors were effective only on AXL-rich cells (Rausch et al., 2017), indicating that the effect is cell-type specific.

Although several studies proclaimed that AXL is a receptor for ZIKV entry *in vitro*, a few reports showed the opposite results. The genetic ablation of AXL has no significant effect on ZIKV entry or ZIKV-mediated cell death in human-induced pluripotent stem cell (iPSC)-derived NPCs or cerebral organoids (Nyboe Andersen et al., 2017; Rausch et al., 2017) reported that Jeg-3 cells that show no detectable AXL expression were highly permissive to ZIKV infection, suggesting that AXL may not be essential for ZIKV infection. This hypothesis is corroborated by an *in vivo* study (Wang Z. Y. et al., 2017). Notably, the AXL receptor supports neural stem cell survival, proliferation and neurogenesis (Ji et al., 2014), and signaling; the AXL also regulates blood–brain barrier (BBB) integrity in the context of viral infections (Miner et al., 2015). Therefore, while blocking AXL may protect against ZIKV infecting or viral replication, perturbation of AXL function may also have multiple adverse consequences. Therefore, the use of the AXL receptor as an idea target for the inhibition of Zika virus infection remains to be confirmed. Efforts to elucidate the molecular mechanism for ZIKV infection, through both targeted TAM receptor knockout studies and unbiased screening for other binding factors that render cells resistant to ZIKV, will lead to the identification of new targets for development of anti-ZIKV therapeutics.

### ZIKV Replication Inhibitors

#### ZIKV Inhibitors Targeting NS2B-NS3 Protease

NS2B-NS3 protease of Zika virus plays an essential role in ZIKV replication and maturation. NS2B-NS3 processes the viral non-structural proteins from the viral polyprotein into individual proteins. NS2B-NS3 is a serine protease that consists of the N-terminal domain of NS3 and a short cofactor from the hydrophilic core sequence of NS2B. Like the NS4A cofactor of the HCV protease, Flavivirus NS3 is inactive without the NS2B co-factor (Erbel et al., 2006).

Three different ZIKV NS2B-NS3 protease (ZIKVpro) constructs have been proposed. First, a covalent G_4_SG_4_ linker peptide between NS2B and NS3 (gZiPro) construct was adopted based on previous West Nile and DENV protease constructs (Lei et al., 2016). The other two constructs include one bivalent protease consisting of two separate polypeptide NS2B and NS3 (bZiPro) (Zhang et al., 2016) and one with its own NS2B C-terminal peptide (TGKR) binding NS2B to NS3 (eZiPro) (Phoo et al., 2016). Remarkably, the single-chain enzyme gZiPro with an artificial linker that is commonly applied for the constructs of other flaviviruses has been widely used for screening inhibitors. Aprotinin, a 58 amino acid bovine trypsin inhibitor, inhibits ZIKV NS2B-NS3 protease with an IC_50_ of 70 nM by blocking the interactions of NS3 and NS2B, as predicted by molecular modeling studies (Table 1; Shiryaev et al., 2017). By using structure-based virtual screening, novobiocin and lopinavir-ritonavir can inhibit ZIKV NS2B-NS3 protease activity by using molecular docking analysis [Figure 3(13–15); Yuan et al., 2017]. Novobiocin, an aminocoumarin antibiotic, inhibited protease activity by highly stable binding with ZIKV NS2B-NS3 protease to diminish its catalytic efficiency (Kirby et al., 1956; Yuan et al., 2017). It can inhibit ZIKV replication with an IC_50_ of 26.12 ± 0.33 μg/ml and CC_50_ of 850.50 μg/ml in Vero cells and an IC_50_ of 38.14 ± 4.53 μg/ml and CC_50_ of 1103.18 μg/ml in Huh-7 cells (Table 3; Yuan et al., 2017). Lopinavir-ritonavir can inhibit protease activity of ZIKV replication with an IC_50_ of 4.78 ± 0.41 μg/ml and CC_50_ of 30.00 μg/ml in Vero cells and an IC_50_ of 3.31 ± 0.36 μg/ml CC_50_ of 32.12 μg/ml in Huh-7 cells (Table 3; Yuan et al., 2017).

**TABLE 3:** Zika virus replication inhibitors.

**Number of chemical structures in Figure 3**	**Inhibitor**	**Testing model**	**Cell lines**	**IC_50_ (μM)**	**CC_50_ (μM)**	**References**
**Inhibitors targeting NS2B-NS3 protease**						
13	Novobiocin	*in vitro*	Vero cells	26.12 ± 0.33	850.50	Kirby et al. (1956), Yuan et al. (2017)
			Huh-7 cells	38.14 ± 4.53	1103.18	
14,15	Lopinavir-ritonavir	*in vitro*	Vero cells	4.78 ± 0.41	30.00	Yuan et al. (2017)
			Huh-7 cells	3.31 ± 0.36	32.12	
16	Phenylacetyl-Lys-Lys-Arg-Gly-Gly-NH2	*in vitro*	–	1.2 ± 0.14	–	Passioura et al. (2014), Phoo et al. (2018), Nitsche et al. (2019)
17	4-guanidinomethyl-phenylacetyl-Lys-Lys-Arg-NH2	*in vitro*	–	1.6 ± 0.14	–	Passioura et al. (2014), Phoo et al. (2018), Nitsche et al. (2019)
18	4-guanidinomethyl-phenylacetyl-Arg-Arg-Arg-4-amidinobenzylamide	*in vitro*	–	1.1 ± 0.07	–	Passioura et al. (2014), Phoo et al. (2018), Nitsche et al. (2019)
19	Hydrolysis product of phenylacetyl-Lys-Lys-Arg-Gly-Gly-NH2	*in vitro*	–	18.4 ± 1.9	–	Passioura et al. (2014), Phoo et al. (2018), Nitsche et al. (2019)
20	Hydrolysis product of 4-guanidinomethyl-phenylacetyl-Lys-Lys-Arg-NH2	*in vitro*	–	5.9 ± 0.55	–	Passioura et al. (2014), Phoo et al. (2018), Nitsche et al. (2019)
21	Bz-[4-(CH2NH2)]Phe-Arg- B(OH)2	*in vitro*	–	0.25	–	Nitsche et al. (2017)
22	Bz-(3-guanidinyl)Phe-Arg- B(OH)2	*in vitro*	–	1.9	–	Nitsche et al. (2017)
23	Bz-(4-guanidinyl)Phe-Arg- B(OH)2	*in vitro*	–	0.83	–	Nitsche et al. (2017)
24	4-tBuBz-(4-guanidinyl)Phe-Arg- B(OH)2	*in vitro*	–	2.1	–	Nitsche et al. (2017)
25	5-amino-1-((4-methoxyphenyl)sulfonyl)-1H-pyrazol-3-yl benzoate	*in vitro*	BznKRR-AMC	1.5 0.1	–	Zhao and Tan (2015)
26	Hydroxychloroquine	*in vitro*	JEG3 cells	–	–	Kumar et al. (2018)
27	Myricetin	*in vitro*	–	1.26	–	Lim et al. (2017), Roy et al. (2017)
28	Apigenin	*in vitro*	–	56.32	–	Roy et al. (2017)
29	Isorhamnetin	*in vitro*	–	15.46	–	Roy et al. (2017)
30	Temoporfin	*in vitro*	A549 cells	1.1 ± 0.1	40.7 ± 0.7	Baell and Holloway (2010), Li et al. (2017d)
31	Nitazoxanide	*in vitro*	A549 cells	15.9 ± 0.9	77 ± 7.2	Baell and Holloway (2010), Li et al. (2017d)
32	BAS 19192837	–	–	–	–	Rohini et al. (2019)
33	Berberine	*in vitro*	–	–	–	Sahoo et al. (2016)
34	Erythrosin B	*in vitro*	A549 cells	0.62 ± 0.12	–	Lee et al. (2017), Li Z. et al. (2018)
35	Luteolin	*in vitro*	–	53 ± 1.3	–	Lim et al. (2017)
36	Astragalin	*in vitro*	–	112 ± 5.5	–	Lim et al. (2017)
37	Rutin	*in vitro*	–	104 ± 2.9	–	Lim et al. (2017)
38	Epigallocatechin gallate	*in vitro*	–	87 ± 1.2	–	Lim et al. (2017)
39	Gallocatechin gallate	*in vitro*	–	99 ± 1.8	–	Lim et al. (2017)
**Inhibitors targeting NS5**						
40	ZINC64717952	*in vitro*	–	–	–	Ramharack and Soliman (2018)
41	ZINC39563464	*in vitro*	–	–	–	Ramharack and Soliman (2018)
42	Baicalein	*in vitro*	Vero cells	0.004	–	Zandi et al. (2012), Moghaddam et al. (2014), Jin et al. (2018), Oo et al. (2018, 2019)
43	DMB213	*in vitro*	Huh-7 cells	5.2	–	Xu et al. (2017)
44	*N*-(4-hydroxyphenyl) retinamide	*in vitro*	Vero cells	1.1 ± 0.4	–	Wang C. et al. (2017)
				1.2 ± 0.1		
45	7DMA	*in vitro*	Vero cells	20 ± 15	>357	Olsen et al. (2004), Zmurko et al. (2016)
				9.6 ± 2.2		
46	Sofosbuvir	*in vitro*	Huh-7 cells Jar human placental choriocarcinoma cells	1 ∼ 5	>200	Lee et al. (2017), Li Z. et al. (2018)
47	F3043-0013	*in vitro*	Vero cells	4.8 ± 2.3	–	Stephen et al. (2016)
48	F0922-0796	*in vitro*	Vero cells	12.5 ± 7.4	–	Stephen et al. (2016)
49	F1609-0442	*in vitro*	Vero cells	17.5 ± 8.4	–	Stephen et al. (2016)
50	F1750-0048	*in vitro*	Vero cells	17.6 ± 3.1	–	Stephen et al. (2016)
51	ZINC50166190	*in vitro*	–	–	–	Singh and Jana (2017)
52	2′-C-Me-UTP	*in vitro*	–	5.78	–	Lu et al. (2017)
53	2′-F-2′-C-Me-UTP	*in vitro*	–	90.76	–	Lu et al. (2017)
54	2′-C-ethynyl-UTP	*in vitro*	–	0.46	–	Lu et al. (2017)
55	3′-dUTP	*in vitro*	–	0.67	–	Lu et al. (2017)
56	Merimepodib (MMPD, VX-497)	*in vitro*	Huh-7 cells	0.6 ± 0.2	>10	Tong et al. (2018)
57	Resiquimod	*in vitro*	CHME3 cells.	–	–	Vanwalscappel et al. (2018)

Recently, Phoo et al. have shown that the glycine-rich artificial linker or “TGKR” peptide could introduce steric hindrance, resulting in the change of the inhibitor-binding mechanism (Phoo et al., 2016). The wide-type NS2B and NS3pro domain are not covalently linked (Kim et al., 2013). bZiPro, which is closer to the native state, has higher activity and is more suitable for inhibitor screening than gZiPro and eZiPro because of its free active site being accessible to substrates or inhibitors (de la Cruz et al., 2011; Phoo et al., 2016; Shannon et al., 2016). Acyl-KR-aldehyde, a dipeptide, can form a covalent bond with the Ser135 residue of NS3 and the KR residues occupy the S1 and S2 sites of NS3 (Li et al., 2017d). Acyl-KR-aldehyde can inhibit the bZiPro construct of NS2B-NS3 protease with an IC_50_ of 208 nM (Table 1; Li et al., 2017d). Nuclear magnetic resonance (NMR) spectroscopy demonstrated that the bZiPro construct of NS2B-NS3 and Acyl-KR-aldehyde can form a stable complex (Li et al., 2017c). The peptidomimetic inhibitors composed of a P1-P4 segment and different P1’ residues, including Phenylacetyl-Lys-Lys-Arg-Gly-Gly-NH_2_, 4-guanidinomethyl-phenylacetyl-Lys-Lys-Arg-NH_2_, 4-guanidinomethyl-phenyl- acetyl-Arg-Arg-Arg-4-amidinobenzylamide, a hydrolysis product of phenylacetyl-Lys-Lys-Arg-Gly-Gly-NH2, and a hydrolysis product of 4-guanidinomethyl-phenylacetyl-Lys-Lys-Arg-NH2 [Figure 3(16–20)], can inhibit ZIKV replication against the NS2B-NS3 protease of ZIKV with an IC_50_ of 1.2 ± 0.14, 1.6 ± 0.14, 1.1 ± 0.07 μM, and 18.4 ± 1.9, 5.9 ± 0.55 μM, respectively (Table 3; Phoo et al., 2018). As non-competitive inhibitors, five macrocyclic peptides can act as ligands with high affinity and can be rapidly isolated for nearly any target by using display screening approaches, such as an mRNA display or phage display (Passioura et al., 2014). These peptides can inhibit bZiPro constructs of NS2B-NS3 protease activity with an IC_50_ from 0.24 to 4.9 μM (Nitsche et al., 2019). AH-D peptide, a 27-mer amphipathic α-helical peptide, protects against lethal ZIKV infection with IC_50_ = 0.012 μM in primary neuronal cells, inhibits ZIKV infection in mouse brains, and preserves BBB integrity (Table 1; Cho et al., 2009; Jackman et al., 2018). The AH-D peptide also significantly reduced viral loads in the serum, brain, spleen, and optical nerve throughout the course of infection (Jackman et al., 2018). Four dipeptidic inhibitors have a C-terminal boronic acid moiety, including Bz-[4-(CH2NH2)]Phe-Arg-B(OH)_2_, Bz-(3-guani-dinyl)Phe-Arg-B(OH)_2_, Bz-(4-guani-dinyl)Phe- Arg-B(OH)_2_, and 4-tBuBz-(4-guanidinyl)Phe-Arg-B(OH)_2_ [Figure 3(21–24)]. These compounds can inhibit ZIKV NS2B-NS3 protease activity with IC_50_ from 0.25 to 2.1 μM (Table 3; Nitsche et al., 2017).

Li Y. et al. have found that a pyrazole ester derivative, 5-amino-1-((4-methoxyphenyl)sulfonyl) -1H-pyrazol-3-yl benzoate [Figure 3(25)], can inhibit ZIKV replication with an IC_50_ of 1.5 μM when benzoyl-Nle-Lys-Arg-Arg-aminomethylcoumarin (BznKRR-AMC) was used as a substrate (Table 3; Li Y. et al., 2018). The benzoyl group of this inhibitor forms a covalent bond with the side chain of catalytic residue S135 to stabilize the closed conformation of the ZIKV bZiPro construct of NS2B-NS3 protease (Li Y. et al., 2018). In addition, a derivative of pyrazole ester, 5-amino-1-((4-methoxyphenyl)sulfonyl)-1H-pyrazol-3-yl benzoate, with an IC_50_ of 0.1 μM can interact in a manner similar to the compound [Figure 3(25)] and strongly inhibit binary ZIKV bZiPro construct of NS2B-NS3 protease (Li Y. et al., 2018). Hydroxychloroquine [Figure 3(26)], a drug already approved and used in pregnancy, can inhibit the bZiPro construct of NS2B-NS3 protease activity with an inhibition constant (Ki) of 92.34 ± 11.91 μM (Table 3; Kumar et al., 2018).

Some natural products from edible plants, like myricetin, can inhibit ZIKV infection with an IC_50_ of 1.26 μM and an inhibitory constant of ZIKV NS2B-NS3 protease activity with *Ki* of 0.77 μM by establishing six hydrogen bonds with four Zika NS3pro residues: Lys73, Asn152, Gln74, and Gly124 [Figure 3(27) and Table 3; Roy et al., 2017]. Apigenin inhibits ZIKV infection with an IC_50_ of 56.32 μM and inhibitory constant *Ki* of 34.02 μM [Figure 3(28) and Table 3; Roy et al., 2017]. Isorhamnetin, also called 3′-Methylquercetin, can inhibit ZIKV infection with IC_50_ of 15.46 μM and *Ki* of 6.22 μM as well as Quercetin with IC_50_ of 2.42 μM and *Ki* of 1.12 μM [Figure 3(29) and Table 3; Roy et al., 2017). Structurally, isorhamnetin is a derivative of quercetin with a very similar molecular structure (Roy et al., 2017). In addition, curcumin [Figure 3(5) and Table 2], a natural phenol with two aromatic rings linked by a heptadiene group, can inhibit ZIKV infection with IC_50_ of 3.45 μM and *Ki* of 2.61 μM, most likely by having bivalent binding sites (Song and Ni, 1998; Roy et al., 2017). The introduction of a C-terminal boronic acid moiety into dipeptidic inhibitors can raise the affinity to target by a 1000-fold (Nitsche et al., 2017). However, curcumin, quercetin, and other flavonoids have shown themselves to be promiscuous inhibitors, e.g., via colloidal aggregation (McGovern et al., 2002; Duan et al., 2015; Tritsch et al., 2015). Curcumin also contains reactive Michael acceptors, and quercetin has a catechol, a well-known PAINS substructure, which might make these compounds less favorable (Mottin et al., 2018).

A total of 2,816 approved and investigational drugs were screened using a high-throughput screening (HTS) assay (Li et al., 2017d). Among these, 23 compounds were confirmed to possess an IC_50_ below 15 μM. Three of them, including temoporfin, niclosamide, and nitazoxanide, could inhibit with an IC_50_ of 1.1 ± 0.1, 12.3 ± 0.6, 15.9 ± 0.9 μM and CC_50_ of 40.7 ± 0.7, 4.8 ± 1.0, 77 ± 7.2 μM in A549 cells, respectively [Figure 3(10,30,31) and Table 3; Baell and Holloway, 2010). Temoporfin was tested in a viremia mouse model and a lethal mouse model, and it was able to inhibit viremia and protect 83% of the mice; the mice that survived did not present any signs of neurological disorder (Li et al., 2017d). These compounds inhibit the interaction between NS3 and the NS2B N-terminal fragment. By using an e-pharmacophore-based virtual screening assay, BAS 19192837 was chosen as a potent Zika NS2B-NS3 protein inhibitor [Figure 3(32)]. However, the experimental data of the IC_50_ about this inhibitor was not shown (Table 3; Rohini et al., 2019).

Berberine, an FDA-approved drug against DENV, has shown high binding affinity of 5.8 kcal/mol, and it binds around the active site of the receptor [Figure 3(33) and Table 3; Sahoo et al., 2016]. Niclosamide, an FDA-approved category B anthelmintic drug for treating worm infections in both humans and domestic livestock, inhibited all three strains of ZIKV, which was measured by intracellular ZIKV RNA levels with IC_50_ values of 1.72 μM in SNB-19 cells (Xu et al., 2016). PHA-690509, an investigational compound that functions as a cyclin-dependent kinase inhibitor (CDKi), inhibited three stains with IC_50_ values of 0.37 μM as measured by intracellular ZIKV RNA levels in SNB-19 cells (Xu et al., 2016). According to experimental results, the mechanism of these two compounds occurs at post-entry stage, likely at the viral RNA replication step (Xu et al., 2016). Suramin [Figure 3(8)], an approved polyanion antiparasitic drug, can be a potential inhibitor of Zika virus complex NS2B/NS3 proteinase with IC_50_ of 47 μM (Table 2; Coronado et al., 2018). Computational analysis showed that suramin suppressed NS2B/NS3 proteinase activity by blocking catalytical Ser135 residue and interacting with the catalytical histidine residue (Coronado et al., 2018). Erythrosin B [Figure 3(34)], a pregnancy category B food additive, inhibited ZIKV replication by targeting NS2B-NS3 proteases with an IC_50_ of 0.62 ± 0.12 μM in A549 cells (Table 3) via a non-competitive mechanism by enzymatic kinetic experiments (Li Z. et al., 2018). Erythrosin B can also inhibit ZIKV RNA synthesis and protein expression in ZIKV-relevant neural progenitor and human placental cells (Li Z. et al., 2018).

Two inhibitors were tested to inhibit NS2B-NS3 protease activity with IC_50_ values of 5.2 and 4.1 μM by blocking the active site of ZIKV NS2B-NS3 protease in docked conformation (Lee et al., 2017). Five polyphenol compounds, including luteolin, astragalin, rutin, epigallocatechin gallate, and gallocatechin gallate from flavone and flavonol, inhibited ZIKV replication with IC_50_ values ranging from 22 ± 0.2 to 112 ± 5.5 μM [Figure 3(35–39) and Table 3; Lim et al., 2017].

#### ZIKV Inhibitors Targeting NS5

Zika virus NS5 is a relative conserved large protein among members of the genus, containing an MTase domain and an RNA polymerase (RdRp) domain connected by a 10 residues linker. ZIKV NS5 can be used as a template for genome replication with an efficiency similar to that of an RNA template (Lu et al., 2017). In one study, ZIKV NS5 could extend an RNA primer annealed to an RNA template in an Mn^2+^-dependent manner, as opposed to Mg^2+^ (Lu et al., 2017). ZINC64717952 and ZINC39563464 can interact with NS5 by enzyme–ligand interactions under virtual conditions to inhibit ZIKV propagation, but no clear data about IC_50_ and CC_50_ have been reported [Figure 3(40,41) and Table 3; Ramharack and Soliman, 2018].

Baicalein [Figure 3(42)], a flavonoid analog, could downregulate ZIKV replication up to 10 h post-infection, while prophylactic effects were evident in pretreated Vero cells with IC_50_ ≈ 0.004 μM and selectivity index (SI) ≈ 105,000 (Table 3; Oo et al., 2019). It also has antiviral activity against DENV, chikungunya virus (CHIKV), and influenza virus (Zandi et al., 2012; Moghaddam et al., 2014; Jin et al., 2018; Oo et al., 2018). DMB213 [Figure 3(43) and Table 3] is a pyridoxine-derived non-nucleoside small-molecule inhibitor with an IC_50_ of 5.2 μM (Xu et al., 2017). This compound inhibits ZIKV by blocking RNA synthesis reactions catalyzed by recombinant ZIKV NS5 polymerase (Xu et al., 2017). A nuclear import inhibitor, *N*-(4-hydroxyphenyl) retinamide [Figure 3(44) and Table 3], can block the interaction between high nanomolar affinity ZIKV NS5 and the host cell importin α/β1 heterodimer (Wang S. et al., 2017). 7-deaza-2′-C-methyladenosine (7DMA) [Figure 3(45)], an inhibitor of hepatitis C virus polymerase (Olsen et al., 2004), could inhibit ZIKV strain MR766 with CC_50_ > 357 μM and an IC_50_ of 20 ± 15 μM in a CPE reduction assay and 9.6 ± 2.2 μM in a virus yield reduction assay in Vero cells (African Green monkey kidney cells; ECACC) (Table 3; Zmurko et al., 2016). *In vivo*, 7DMA also reduced viremia (between day 3 and 8 post infection) and delayed virus-induced morbidity and mortality in AG129 (IFN-α/β and IFN-γ receptor knockout) mice infected with ZIKV (Zmurko et al., 2016). Sofosbuvir [Figure 3(46)], an FDA-approved nucleotide polymerase inhibitor, can efficiently inhibit replication and infection of several ZIKV strains, including African and American isolates, with IC_50_ values of 1–5 μM and CC_50_ > 200 μM in Huh-7 and Jar human placental choriocarcinoma cells (Table 3; Bullard-Feibelman et al., 2017). In addition, oral treatment with sofosbuvir also protected against ZIKV-induced death in 5-week-old C57BL/6J mice (Li Z. et al., 2018).

Using virtual screening, Stephen et al. identified four top-scoring ligands from the 28,341 compounds, including F3043-0013, F0922-0796, F1609-0442, and F1750-0048, against ZIKV with IC_50_ values of 4.8 ± 2.3, 12.5 ± 7.4, 17.5 ± 8.4, and 17.6 ± 3.1 μM, respectively, by plaque reduction assay (PRA) [Table 3 and Figure 3(47–50); Stephen et al., 2016]. These compounds may cooperatively interact with the hydrophobic binding pocket, extending to the S-adenosyl homocysteine (SAH) binding pocket (Stephen et al., 2016). Based on the modeling of the catalytic domain of ZIKV RNA-dependent RNA polymerase (RdRpC), Ligand 6 [(S)-2-(3-hydroxyphenyl)-*N*-(1,2,3,4-tetrahydronaphthalen-1yl)acetamide] (ZINC50166190) was chosen as potentially having inhibitory activity against ZIKV RdRpC protein by acting as a GTP-nucleotide analog to prevent initiation of ZIKV RNA polymerization [Figure 3(51) and Table 3; Singh and Jana, 2017). Some ribonucleotide 5′-triphosphate analogs like 2′-C-Me-UTP, 2′-F-2′-CMe-UTP, 2′-C-ethynyl-UTP, and 3′-dUTP inhibit ZIKV replication with IC_50_s of 5.78, 90.76, 0.46, and 0.67 μM [Figure 3(52–55) and Table 3], respectively, as measured in the presence of 1 μM competing UTP by an alternative non-radioactive coupled-enzyme assay (Lu et al., 2017). One of the major inhibitory mechanisms of ribonucleotide 5′-triphosphate against ZIKV infection is its direct termination of viral RNA polymerases (Lu et al., 2017). Merimepodib (MMPD, VX-497) [Figure 3(56)], a potent inhibitor of inosine-5′-monophosphate dehydrogenase (IMPDH), inhibited ZIKV RNA replication with an IC_50_ of 0.6 ± 0.2 μM and IC_90_ of 1.0 ± 0.2 μM in Huh7 cells. In the cytotoxicity assay, the CC_50_ was determined to be >10 μM, which resulted in the selective index > 17 μM (Table 3; Tong et al., 2018). TLR7/8 agonist R848 (resiquimod) (toll-like receptor (TLR) agonists) inhibited ZIKV RNA synthesis in CHME3, a transformed microglial cell-line [Figure 3(57) and Table 3; Vanwalscappel et al., 2018).

### Other Small-Molecule Inhibitors With Undefined Mechanisms

AV-C(1-(2-fluorophenyl)-2-(5-isopropyl-1,3,4-thiadiazol-2-yl)-1,2-dihydrochromeno[2,3-c]pyrrole-3,9-dione) [Figure 3(58)], an interferon-activating agonist of the TRIF pathway, inhibited replication of ZIKV (IC_90_) of 5.815 μM by activating innate- and interferon-associated responses, and the CC_50_ values are well below dosages observed to induce detectable cytotoxicity of THF cells (Table 4; Pryke et al., 2017). Along with Selenium, a free-form amino acid sequence (FFAAP) comprising glycine, cystine, and a glutamate source inhibited ZIKV replication with an ED_90_ (effective dose at which 90% of a dose of Zika virus was inhibited) of 2.5 mM in human JEG-3 cells and 4 mM in Vero cells (Table 1; Vasireddi et al., 2019). Histone H3K27 methyltransferases EZH2 and EZH1 (EZH2/1) can suppress gene transcription via propagation of repressive H3K27me3-enriched chromatin domains (Margueron and Reinberg, 2011; Di Croce and Helin, 2013; Kim et al., 2015). GSK926 [Figure 3(59)], an inhibitor of histone methyltransferases EZH2/1 (Verma et al., 2012), can suppress not only DNA viruses, like herpes simplex virus (HSV) and human cytomegalovirus (adenovirus) infection, but also an RNA virus, like ZIKV replication, in human foreskin fibroblast (HFF) cells (Table 4; Arbuckle et al., 2017). However, no IC_50_ was reported. T-705 (favipiravir) [Figure 3(60)], a broad-spectrum antiviral with effects against many viruses, and a structural analog, T-1105, inhibited ZIKV strain SZ01 with an IC_50_ of 110.9 ± 13.1 and 97.5 ± 6.8 μM, respectively, and CC_50_ > 3000 μM in Vero cells (Table 4; Cai et al., 2017). Emricasan [Figure 3(61)], a pan-caspase inhibitor, inhibited ZIKV-induced increases in caspase-3 activity with IC_50_ values of 0.13–0.9 μM in SNB-19 cells against three ZIKV strains: FSS13025 (2010 Cambodian strain), MR766 (1947 Ugandan strain), and PRVABC59 (2015 Puerto Rican strain; Table 4; Xu et al., 2016). In addition, in Caspase-3/7 assay, Emricasan also reduced the total number of active (cleaved) caspase-3-expressing forebrain-specific hNPCs (human cortical neural progenitor cells) in both the monolayer and 3-dimensional organoid cultures infected by ZIKV FSS13025 strain (Xu et al., 2016). Two halogenated chrysins, FV13 and FV14 [Figure 3(62,63)], inhibited ZIKV infection with an IC_50_ of 1.65 ± 0.86 and 1.39 ± 0.11 μM in LLC/MK2 cells, respectively (Suroengrit et al., 2017). The CC_50_s to the LLC/MK2 cell-based system of FV13 and FV14 were 44.28 ± 2.90 and 42.51 ± 2.53 μM, respectively (Table 4; Suroengrit et al., 2017). The investigations into the mechanism of these two halogenated chrysin actions suggested multiple targets, but maximal efficiency was achieved with early post-infection treatment (Suroengrit et al., 2017). Compound 1 was confirmed with an inhibitor of ZIKV-induced cytopathic effect (CPE) with an IC_50_ of 5.95 μM and a CC_50_ of 100 μM in human fetal neural stem cells (NSCs) [Figure 3(64) and Table 4; Bernatchez et al., 2018). PKI 14-22 (PKI) [Figure 3(65)], a PKA inhibitor, could act as a potent inhibitor of Asian/American and African lineages of ZIKV replication with an IC_50_ of about 20 μM in endothelial cells and astrocytes by minimal cytotoxicity (Table 4; Cheng et al., 2018). Cavinafungin [Figure 3(66)], targeting ER signal peptidase by binding on SEC11, inhibited ZIKV replication with an IC_50_ of 150 nM and CC_50_ of 1650 nM in A549 cells (Table 4; Estoppey et al., 2017). Manidipine and cilnidipine, voltage-gated Ca^2+^ channel (VGCC) inhibitors, could inhibit ZIKV infection by 100% with no plaque formation observed at a concentration of 10 μM in Vero cells (Wang S. et al., 2017). According to accumulating data, benidipine hydrochloride, pimecrolimus, and nelfinavir mesylate can also inhibit ZIKV replication with a concentration of 10 μM in Vero cells [Table 4 and Figure 3(67–69); Wang S. et al., 2017]. Five 2′-C–methylated derivatives of nucleoside analog [nucleosides with a methyl moiety at the 2′-C position of the ribose ring, including 2′-CMA,7-deaza-2′-CMA, 2′-CMC, 2′-CMG, and 2′-CMU [Figure 3(70–74)], can reduce the viral titer with an IC_50_ of 5.26 ± 0.12 μM for 2′-CMA, 8.92 ± 3.32 μM for 7-deaza-2′-CMA, 10.51 ± 0.02 μM for 2′-CMC, 22.25 ± 0.03 μM for 2′-CMG, and 45.45 ± 0.64 μM for 2′-CMU in Vero cells (Table 4; Eyer et al., 2016). All these nucleoside analogs showed weak or no cytotoxic effects at a concentration of 100 μM on cell proliferation (Eyer et al., 2016). NGI-1 [Figure 3(75)], an aminobenzamide-sulfonamide compound targeting both oligosaccharyltransferase OST isoforms, can block viral RNA replication significantly to inhibit viral particle formation with an IC_50_ of 2.2 μM in HEK293 cells (Table 4; Puschnik et al., 2017). Nordihydroguaiaretic (NDGA) and its methylated derivative, tetra-*O*-methyl nordihydroguaiaretic acid (M4N), disturbed lipid metabolism and sterol regulatory element-binding proteins (SREBP) [Figure 3(76–77)], thereby inhibiting ZIKV PA259459 isolated from a patient with an IC_50_ of 9.1 μM and SI of 17.8 and IC_50_ of 5.7 μM and SI of 187.9, respectively (Table 4; Merino-Ramos et al., 2017). Obatoclax, SaliPhe, and gemcitabine affected ZIKV-mediated transcription [Figure 3(78–80) and Table 4], translation, and posttranslational modifications as well as metabolic pathways, by different mechanisms of action, inhibiting ZIKV infection at non-cytotoxic concentrations (Kuivanen et al., 2017). ZMP STE24, when complexed with proteins of the interferon-induced transmembrane protein (IFITM) family by co-immunoprecipitation studies, inhibited ZIKV viral titer in T98-G cells (Table 1; Fu et al., 2017). PHA-690509 [Figure 3(81)], an investigational compound that functions as a CDKi, inhibited three ZIKV stains with IC_50_ values of 0.37 μM, as measured by intracellular ZIKV RNA levels in SNB-19 cells (Table 3; Xu et al., 2016).

**TABLE 4:** Other small-molecule inhibitors with undefined mechanisms.

**Number of chemical structures in Figure 3**	**Inhibitor**	**Testing model**	**Cell lines**	**IC_50_ (μM)**	**CC_50_ (μM)**	**References**
58	AV-C	*in vitro*	THF cells	–	–	Pryke et al. (2017)
59	GSK926	*in vitro*	HFF cells	–	–	Verma et al. (2012)
60	T-705	*in vitro*	Vero cells	110.9 ± 13.1	>3000	Cai et al. (2017)
61	Emricasan	*in vitro*	SNB-19 cells	0.13 ∼ 0.9	–	Xu et al. (2016)
62	FV13	*in vitro*	LLC/MK2 cells	1.65 ± 0.86	44.28 ± 2.90	Suroengrit et al. (2017)
63	FV14	*in vitro*	LLC/MK2 cells	1.39 ± 0.11	42.51 ± 2.53	Suroengrit et al. (2017)
64	Compound 1	*in vitro*	Human fetal neural stem cells	5.95	100	Bernatchez et al. (2018)
65	PKI 14-22	*in vitro*	Endothelial cells	20	–	Cheng et al. (2018)
66	Cavinafungin	*in vitro*	A549 cells	150	1650	Estoppey et al. (2017)
67	Benidipine hydrochloride	*in vitro*	Vero cells	–	–	Wang S. et al. (2017)
68	Pimecrolimus	*in vitro*	Vero cells	–	–	Wang S. et al. (2017)
69	Nelfinavir mesylate	*in vitro*	Vero cells	–	–	Wang S. et al. (2017)
70	2′-CMA	*in vitro*	Vero cells	5.26 ± 0.12	–	Eyer et al. (2016)
71	7-deaza-2′-CMA	*in vitro*	Vero cells	8.92 ± 3.32	–	Eyer et al. (2016)
72	2′-CMC	*in vitro*	Vero cells	10.51 ± 0.02	–	Eyer et al. (2016)
73	2′-CMG	*in vitro*	Vero cells	22.25 ± 0.03	–	Eyer et al. (2016)
74	2′-CMU	*in vitro*	Vero cells	45.45 ± 0.64	–	Eyer et al. (2016)
75	NGI-1	*in vitro*	HEK293 cells	2.2	–	Puschnik et al. (2017)
76	NDGA	*in vitro*	Vero cells	9.1	–	Merino-Ramos et al. (2017)
77	M4N	*in vitro*	Vero cells	5.7	–	Merino-Ramos et al. (2017)
78	Obatoclax	*in vitro*	RPE cells	0.04 ± 0.01	2.6 ± 0.4	Kuivanen et al. (2017)
79	SaliPhe	*in vitro*	RPE cells	0.05 ± 0.02	>10	Kuivanen et al. (2017)
80	Gemcitabine	*in vitro*	RPE cells	0.01	>10	Kuivanen et al. (2017)
81	PHA-690509	*in vitro*	SNB-19 cells	0.37	–	Xu et al. (2016)

## Strategies for Developing Small-Molecule ZIKA Inhibitors

The development of anti-ZIKV drugs requires effective strategies. For example, using existing drug libraries to screen drug molecules that inhibit new targets is an effective method to develop new drugs (Yang et al., 2017; Kumar et al., 2018). In silicon-based drug modeling, it is another economical and useful strategy to identify candidate drugs in a short amount of time (Pal et al., 2017; Kumar et al., 2018). In addition, virtual screening and electronic pharmacokinetic modeling also facilitate the discovery of effective drug molecules (Rohini et al., 2019). First, the virtual screening method based on electronic pharmacodynamics was adopted to screen effective inhibitors of ZIKV NS2B-NS3 protein from the ASINE database (including 467,802 molecules) (Rohini et al., 2019). Then, the complexes of known NS2B-NS3 protein and its inhibitor were used to establish a five-featured pharmacophore hypothesis, ADDRR, which consists of one hydrogen bond acceptor (A), two hydrogen bond donors (D), and two aromatic rings (R) (Rohini et al., 2019). The pharmacophore model was verified by enrichment analysis before the virtual screening process (Rohini et al., 2019).

Active development of screening methods to assess the antiviral activity of compounds is a key step in the discovery of new drugs (Ekins et al., 2016). Virus replication relies on cellular mechanisms, which means that *in vitro* experiments use host cells for culture and virus replication. Since ZIKV can infect many different cells, multiple cell lines should be used to study ZIKV infection. The screening of effective drugs using multiple cells provides a good framework for drug discovery (Barr et al., 2016). Other strategies include RNA interference, long non-coding RNAs, miRNAs, interfering peptides, and compounds targeting viral RNA (Han and Mesplede, 2018), underexplored building blocks, and elements introducing into medicinal chemistry (Nitsche et al., 2017).

## Conclusion

As an arthropod-borne single-stranded positive RNA virus, ZIKV utilizes a number of host viral proteins and cellular components to accomplish its replication cycle, including the steps of viral entry, genomic replication, structural and non-structural protein processing, assembly, and budding of virions. Such actions result in a series of congenital abnormities like Guillain–Barré syndrome in adults, microcephaly in newborns, and fetal demise during pregnancy (Dick et al., 1952), and the viral and host proteins involved in the virus life cycle can serve as targets for development of small-molecule ZIKV inhibitors. For example, the ZIKV E protein is responsible for the binding of the virus to host cell receptors and mediating viral entry into the host cell; therefore, some small molecule inhibitors targeting the ZIKV E protein are effective in inhibiting virus attachment and entry (Byrd et al., 2013; Fernando et al., 2016; Oo et al., 2019). AXL expressed on human glial cells can permit ZIKV binding and entry into the host glial cells (Nowakowski et al., 2016; Meertens et al., 2017) and small molecule compounds targeting AXL may be effective in inhibiting ZIKV infection (Rausch et al., 2017). However, any compounds targeting host proteins may affect their normal functions and cause adverse effects.

Study has shown that, since the stem region of the ZIKV E protein has high sequence similarity to that of other flavivirues, such as DENV and yellow fever virus (YFV), the ZIKV inhibitor targeting this region is also highly effective against DENV and YFV infection (Yu et al., 2017). Therefore, it is essential to develop small molecule compounds with broad flavivirus inhibitory activity. Another important strategy is to develop small molecule ZIKV inhibitors targeting the different steps of ZIKV replication cycle with a synergistic antiviral effect when they are used in combination.

Numerous cases of ZIKV sexual transmission have been reported during recent ZIKV outbreaks, and studies have shown that ZIKV also replicates in human prostate cells (Spencer et al., 2018). However, little is known about what viral protein(s) and host factor(s) are involved in this event. Therefore, it is essential to identify these proteins as targets for development of small-molecular inhibitors for preventing sexual transmission of ZIKV.

With the increasing understanding of viral protein structure, tremendous progresses have been made in structure-based discovery of inhibitors targeting the structure and non-structure protein of ZIKV, such as the E protein, three NS2B-NS3 proteinase constructs and helicase, NS5 methyltransferase and polymerase. Several series of small-molecule ZIKV inhibitors targeting these proteins have been reported. However, most of them were tested *in vitro* while only a small percentage of these compounds have been evaluated in animal models *in vivo*, and very few have advanced into clinical trials. Therefore, further studies should focus on exploiting novel strategies to identify new anti-ZIKV compounds, elucidating their mechanisms of action, improving the efficacy of anti-ZIKV compounds, and evaluating the *in vivo* efficacy and safety of these compounds in suitable animal models and patients. Further development of small-molecule ZIKV inhibitors with high-efficiency and low toxicity will bring promise for clinic treatment of ZIKV infection and related diseases in the near future.

## Author Contributions

LW, RL, YG, YL, XD, RX, YZ, and FY drafted the manuscript. TY, SJ, and FY revised and edited the manuscript.

## Conflict of Interest

The authors declare that the research was conducted in the absence of any commercial or financial relationships that could be construed as a potential conflict of interest.
